# Integrating System Dynamics and Action Research: Towards a Consideration of Normative Complexity: Comment on "Insights Gained From a Re-analysis of Five Improvement Cases in Healthcare Integrating System Dynamics Into Action Research"

**DOI:** 10.34172/ijhpm.2023.7582

**Published:** 2023-05-21

**Authors:** Rik Wehrens, Lieke Oldenhof, Marjolijn Heerings, Violet Petit-Steeghs, Sander van Haperen, Roland Bal, Trisha Greenhalgh

**Affiliations:** ^1^Erasmus School of Health Policy & Management, Erasmus University, Rotterdam, The Netherlands; ^2^University of Oxford, Oxford, UK

**Keywords:** Complexity, Action Research, System Dynamics, Mixed Methods, Pragmatism, Values

## Abstract

Holmström and co-authors argue for the value of integrating system dynamics into action research to deal with increasing complexity in healthcare. We argue that despite merits, the authors overlook the key aspect of normative complexity, which refers to the existence of multiple, often conflicting values that actors in healthcare systems have to pragmatically develop responses to in their daily practices. We argue that a better theoretical and empirical understanding of the multiplicity of values and how actors deal with value conflicts in daily practices can enrich discussions about complexity in healthcare. We introduce the alternative methodology of ‘value exnovation’ for action researchers to broaden the scope of system-based thinking and action research in healthcare.

 Holmström and co-authors argue for integrating system dynamics into action research to help ensure that outcomes of healthcare improvement projects are more “useful, comprehensive, and robust for the client organization than when applying either approach in isolation.”^1^ Through a reanalysis of five improvement cases, they show that increasing complexity in healthcare necessitates a move away from top-down improvement trajectories as these are insufficiently calibrated to local needs and circumstances, thus reducing the likelihood of sustained uptake of such improvements. According to the authors, integrating methods derived from system dynamics (such as causal loop diagrams) into change-oriented action research offers a productive mixed-methods process that allows engaged researchers and practitioners to build on the strengths of both approaches. The “high level of engagement and ownership” among participants attributed to action research, combined with the “causal rigor, consistency, and reality checks” provided through the application of system dynamics, is thus argued to converge into more than the sum of its parts.

 The paper has many merits. First, the authors convincingly describe how the combination of action research and system dynamics allows researchers and practitioners to better ‘work with’ a complex healthcare system, building on both methods’ specific strengths.^2^ Secondly, the authors discuss the iterative processes of their research and how they abductively analyzed and compared findings. In a domain that is still predominantly built around ideals of randomized controlled trials and other rationalist approaches (such as cost-effectiveness analysis),^3^ research methods that emphasize flexibility, tailoring, creativity, and (joint) interpretation of results are highly needed. Thirdly, the article serves as an example of how a secondary analysis of various case studies can contribute to developing new methodological and theoretical insights. The paper therefore illustrates how a combination of methodological approaches, combined with analytical rigor and sensitivity towards problems experienced in the practice of healthcare organisations can lead to both practically useful interventions *and *new analytical insights.

## Towards a Consideration of Normative Complexity in Healthcare

 Holmström et al consider healthcare to be complex due to several factors: multi-professional staff and a variety of patient care pathways, time pressure and minimal margins for errors, and tension between hierarchical power and the power of the professions.^1^ Through this focus the authors overlook a key aspect when it comes to complexity in healthcare: *normative complexity. *According to Cribb et al^4^ who recently coined this term, normative complexity relates to different perspectives on “what is valuable in healthcare” (p. 85). Normative complexity thus refers to the existence of multiple, often conflicting values that actors in complex healthcare systems have to pragmatically develop responses to in their daily practices.^5,6^

 Holmström et al are not unique in overlooking normative complexity. Despite the increasing uptake of complexity science in healthcare research,^7^ the focus of most authors centers on studying healthcare as a set of dynamic processes characterized by unpredictability, path-dependency, emergence, and phase transitions.Most discussions of complexity in healthcare therefore focus primarily on what Cribb et al^4^ call explanatory complexity, with normative complexity either absent or (at best) implicit (eg, through a focus on processes of sense-making) but under-theorized.

 In this response, we utilize the paper by Holmström et al to show that attention for normative complexity can add value to system dynamics. We argue that a better theoretical and empirical understanding of the multiplicity of values and how actors deal with value conflicts in daily practices can enrich discussions about complexity in healthcare.

## Normative Complexity: A Pragmatist Approach

 We build on the work of various pragmatist scholars outside the realm of complexity science who have already shown how multiple, conflicting values are part and parcel of healthcare systems.^8^

 A pragmatist value approach does not treat values as abstract principles, but places values firmly inside mundane healthcare practices, thereby building on the work of early pragmatists such as Dewey.^9^ As Dussauge et al^8^ note, “values should be seen as always already constituted in practices, not as static entities which exist outside of action” (p. 19). As such, values can be defined as emergent qualities rooted in the demands of concrete practices, such as care giving, strategy making or managerial decision-making.However, this does not mean that practices merely bring to the fore already existing values. Practices can also be constitutive of value-making. It is in daily healthcare practices that value (or “worth”) is created in situ: through interactions between human actors (patients, healthcare professionals, managers, etc) and non-human actors (electronic patient records, decision support systems, architecture).

 The fact that multiple values are embedded in healthcare practices does not necessarily make them complex. After all, multiple values can peacefully co-exist or values can sometimes be ‘optimized.’ Normative complexity especially arises in three situations, which we consider in turn below.

 First, when values such as quality, safety, personalization, efficiency, patient-centeredness, and empowerment are interpreted and operationalized in different ways. A single value such as patient safety can already create normative complexity when multiple interpretations (‘strict’ versus ‘lenient’) and operationalizations (eg, conflicting safety protocols) exist. Furthermore, multiple values can clash — for example patent safety may clash with other values such as autonomy, personalized care or patient choice. For example, arranging independent living situations for people with serious mental illness gives them autonomy on how they manage their household and who can enter their homes, but also provides safety issues when signs of deterioration or abuse go unnoticed as service users do not allow professionals to enter. Here values of autonomy, person-centered care and safety uncomfortably clash.^10^

 Second, when some values are more firmly institutionally embedded than others, thus making it difficult to act on less institutionalized values. For example, in home care services, values such as efficiency are embedded in models of accounting in which care is Taylorized into different physical care tasks for which a specific amount of time (in minutes) is available. Such contexts render invisible values that cannot be accounted for within this model, such integrated or social care.^11^

 Third, when values change over time. Emergence and dynamism are recognized in complexity science, showing non-linear interactions between different subsystems. Normative complexity is similarly emergent and dynamic. Due to the emergence of new care paradigms and policy discourses, values are reconfigured and differently prioritized over time. For example, market paradigms of healthcare may gradually be replaced or layered with communal healthcare paradigms.^12^

 Together, these layers of normative complexity which take into account the different perspectives on what is considered valuable in health and social care have potential to further enhance explanatory forms of complexity as advocated by Holmström et al. When focusing on the normative question what ‘should’ matter in healthcare, it becomes especially relevant to zoom in on the ways healthcare practitioners and action researchers can actually deal with normative complexity.

## Dealing With Normative Complexity: The “Exnovation” Role of the Action Researcher

 From a normative complexity perspective, it is unlikely that healthcare practitioners will fundamentally ‘converge’ or ‘agree’ on one-best way of dealing with wicked problems as Holmström et al suggest. This would deny the existence of value conflicts that give rise to wicked problems in the first place. We therefore take issue with the suggested role of action researchers as facilitators of a process in which stakeholders — after an initial phase of divergent thinking — converge on a mutually developed point of reference.^1^

 Instead of convergence facilitators, we think it would be more productive to view action researchers as ‘value exnovators’ that focus on excavating implicit (conflicting) values in practices and strategies to deal with these values (adapted from Mesman^13^). This is not an easy thing to do, as underlying values of a problem cannot be easily ‘read’ or ‘inventorized’ with a survey. By looking at particular verbal and material clues, however, exnovation is possible. These clues can be found in recurring vocabulary that practitioners use which reflects what they think is valuable or important, and routines and strategies that practitioners employ to deal with conflicting values (cf. Oldenhof et al^6^). Reflecting on this vocabulary and strategies together with practitioners can open up new action repertoires, ways of thinking and processes of learning. Such an approach has parallels to what Schön and Rein called ‘frame-reflective awareness’ — an orientation which seeks to understand and take account of the different values being brought into play by other stakeholders, even when one does not share those perspectives or values.^14^

 A potential downside of the role of action researchers as ‘value exnovators’ could be that detailed qualitative investigations can be time-consuming. Moreover, managers and policy-makers could consider the exposure of value conflicts as unproductive as they would prefer more solution-oriented results. But while system-based approaches (such as in silico simulations) may seem more efficient, they always remain model-based simplifications of a more normatively complex social world, and thus always require additional translation efforts to become actualized in practice. Whereas qualitative investigations thus may be more time-consuming in the initial stages of data collection and analysis, the additional work of system-based approaches resides in the ongoing translation work that needs to be conducted to implement the proposed (simulated) interventions. Moreover, the perceived ‘unproductivity’ for which action research can be criticized is usually grounded in a rather narrow, instrumentalist perspective on utility, in which usefulness is reduced to offering ‘solutions’ [cf. Zuiderent-Jerak et al^15^]. This reading seems to neglect the added value that frame reflection and the identification of action repertoires can have more indirectly and longitudinally.

## Conclusion

 In sum, Holmström et al describe an innovative mixed methods research approach that allows researchers and practitioners to get a better grasp on the way interventions and improvement projects can play out in the dynamics of complex health systems. We believe they could extend this approach by taking into account the various value conflicts and (mis)alignments that take place in health and care practices. We have introduced the notion of normative complexity and the alternative methodology of ‘value exnovation’ for action researchers to broaden the scope of system-based thinking and action research in healthcare. There seems to be much potential to further combine different approaches to system-based action research. Working towards analyses that combine simulation-based approaches to complexity with ways to ethnographically tease out dimensions of normative complexity might turn out to be particularly productive in understanding *and *intervening in the complex challenges healthcare practitioners are confronted with.

## Ethical issues

 Not applicable.

## Competing interests

 Authors declare that they have no competing interests.
